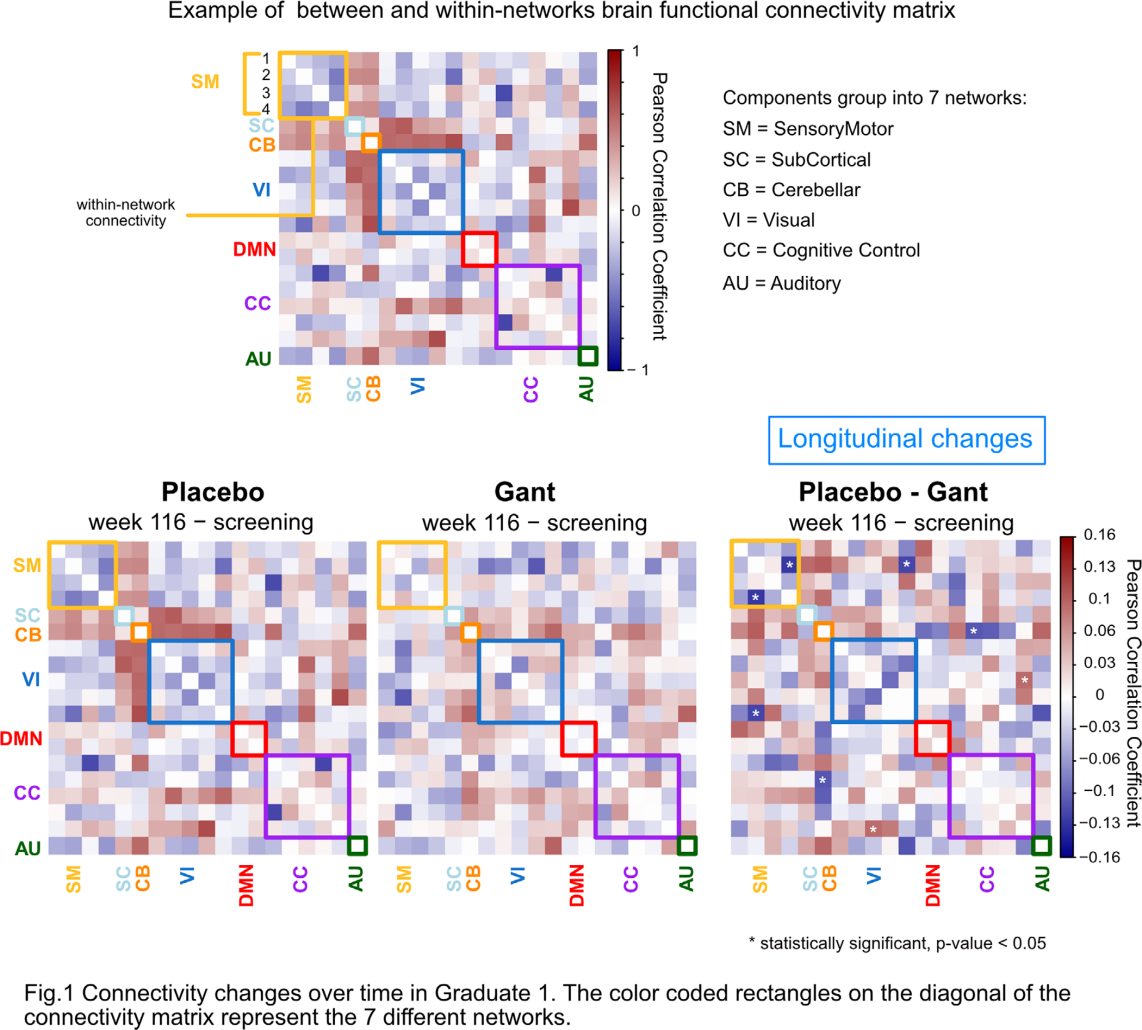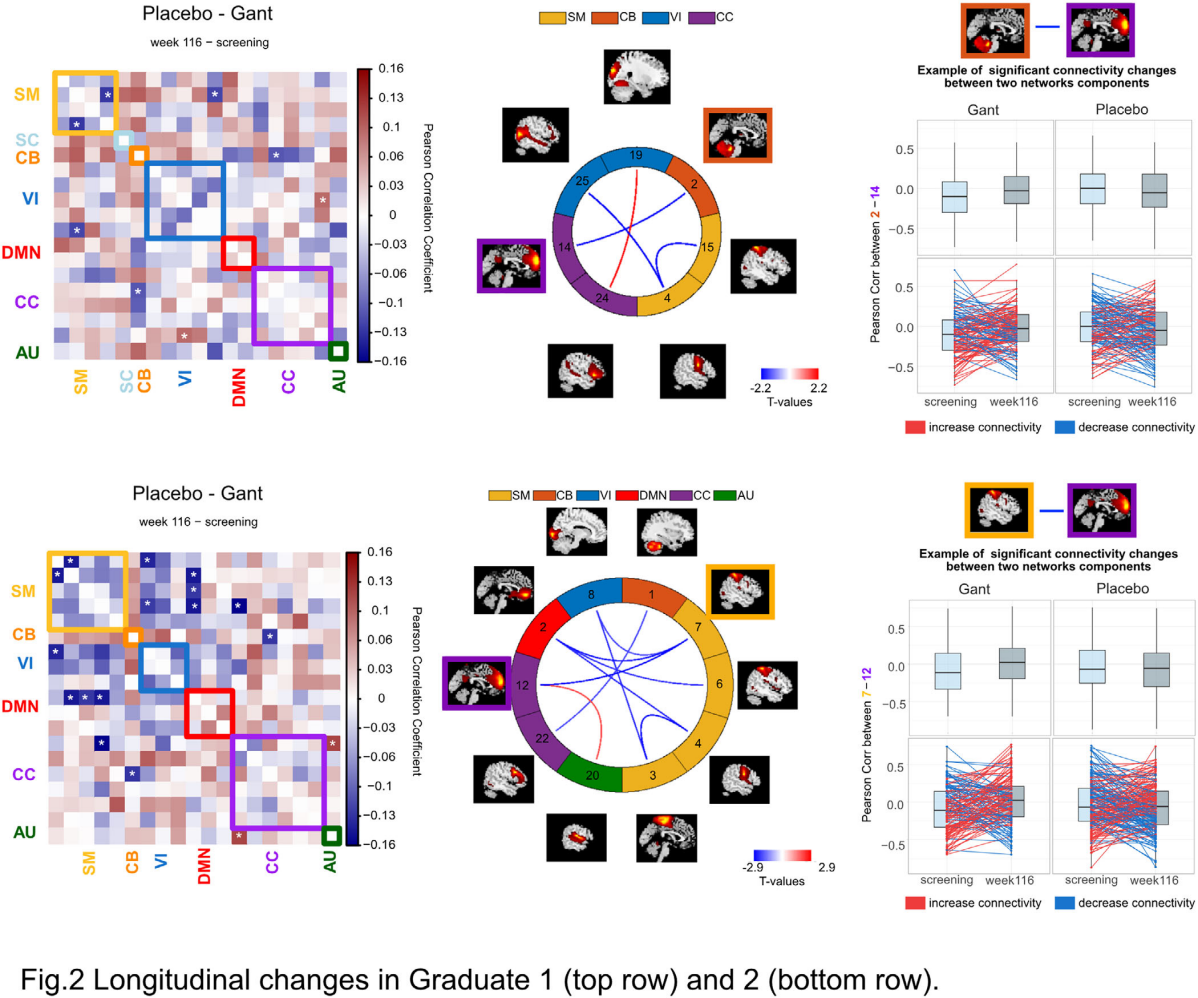# The effect of gantenerumab treatment on brain functional connectivity

**DOI:** 10.1002/alz.094926

**Published:** 2025-01-09

**Authors:** Susanna Gobbi, Gregory Klein, Martijn van den Heuvel, Stefano Magon

**Affiliations:** ^1^ Roche Pharma Research and Early Development, Neuroscience and Rare Diseases Biomarkers, Basel Switzerland; ^2^ Center for Neurogenomics and Cognitive Research, Vrije Universiteit and Amsterdam University Medical Center, Amsterdam Netherlands

## Abstract

**Background:**

Changes in brain functional connectivity obtained from resting state MRI (rs‐fMRI) have been found to be associated with cognitive decline and neurodegeneration in clinical trial participants with Alzheimer’s disease (AD). This study investigates whether and how this technique can be used in AD clinical trials to monitor treatment effects. Specifically, we analyzed changes in brain functional connectivity over a period of 116 weeks in participants with AD treated with gantenerumab, an investigational anti‐amyloid beta monoclonal antibody. Although this drug did not meet the primary clinical endpoints, some significant associations were found with exploratory biomarkers.

**Method:**

We analyzed rs‐fMRI of participants with MCI due to AD and mild AD at screening and week 116 collected in a subgroup of the randomized, double‐blind, pivotal, Phase III trials GRADUATE I (273 patients, 52% female, 50% placebo) and GRADUATE II (335 patients, 59% female, 57% placebo) (NCT03444870, NCT04374253). Using group Independent Components Analysis (GIFT, Calhoun et al., 2001), we extracted 25 resting states components in each clinical trial and grouped them into 7 networks (Sensory Motor, Cerebellar, Visual, SubCortical, Auditory, Default Mode and Cognitive Control). Using a linear model, we investigated changes in between‐ and within‐networks brain connectivity at screening and week 116 in gantenerumab and placebo participants. The components‐wise Pearson’s correlation (Z‐Fisher score) was used as the dependent variable, group (gantenerumab vs placebo), visit (screening vs week 116) and the interaction term group‐by‐visit as covariates of interest, while controlling for sex, age and scanner magnetic field strength (1.5T vs 3T). This analysis was exploratory in nature and no adjustment for multiple components‐wise comparisons was applied.

**Result:**

In both GRADUATE I and II, we found a significant group‐by‐visit effect (p < 0.05). Specifically, we found decreasing within‐ and between‐networks functional connectivity in the placebo group at week 116 compared to screening. Conversely, the gantenerumab group reported stable or higher connectivity at week 116 compared to screening (Fig. 1‐2).

**Conclusion:**

These results suggest a potential relationship between amyloid removal and changes in brain functional connectivity. Due to the exploratory nature of this study further investigation is needed to confirm these findings.